# Editorial: The current role of allergy in otolaryngological disorders

**DOI:** 10.3389/falgy.2024.1498340

**Published:** 2024-10-10

**Authors:** Luca Giovanni Locatello, David Lobo, Alberto Maria Saibene, Carlo Pucillo

**Affiliations:** ^1^ Department of Otorhinolaryngology, Academic Hospital “Santa Maria della Misericordia”, Azienda Sanitaria Universitaria Friuli Centrale, Udine, Italy; ^2^ Otolaryngology Department, Hospital Universitario Marques de Valdecilla, Santander, Spain; ^3^ Valdecilla Biomedical Research Institute, IDIVAL, Santander, Spain; ^4^ Otolaryngology Unit, Department of Health Sciences, Santi Paolo e Carlo Hospital, Università Degli Studi di Milano, Milan, Italy; ^5^ Immunology Section, Department of Medicine (DAME), University of Udine, Udine, Italy

**Keywords:** sinusitis, allergy, rhinology, immunology, otorhinolarygology

**Editorial on the Research Topic**
The current role of allergy in otolaryngological disorders

Historically, Otorhinolaryngology has been closely linked with the immune system since its inception. Surgically treated disorders of the mucosal-associated lymphoid tissue (MALT, in the form of palatine tonsils and adenoids), or the sinonasal microenvironment (presenting as CRS, chronic rhinosinusitis with and without nasal polyps) are in truth immunopathological conditions (1, 2). It is well accepted that procedures such as adenotonsillectomy or endoscopic sinus surgery will still be widely performed in the future. At the same time, our understanding of the interplays between the immune system and the ear, nose, and throat continues to deepen with advances in molecular biology. For example, recent research has identified a subset of peptidase inhibitor 16 (PI16)-expressing fibroblastic reticular cells (FRCs) in human tonsils (3). The population of PI16-FRCs is not present in the pediatric patients and it increases in the aging process; it was shown that this is the cellular subset that undergoes the most profound remodeling as it shapes the tonsillar microenvironment for efficient lymphocyte activation and differentiation (3).

As Editors of the Research Topic “*The Current Role of Allergy in Otolaryngological Disorders*”, published in the present Journal, we have the privilege of presenting several insightful manuscripts that shed new light on the subject. A notable study by Barreto et al. have used a longitudinal cohort of poly-sensitized children with seasonal allergic rhinitis (AR), in order to identify the allergen most likely to be used in nasal provocation testing (NPT). Determining the exact inflammatory trigger is important to maximize the efficacy of allergen immunotherapy (AIT). The authors have therefore analyzed an extensive amount of clinical and patient-reported data, including those collected by an e-diary smartphone application. After applying logistic regression, they discovered that a combination index of specific IgE activity towards Phl p 5 and Cyn d 1 could predict positive NPT results to grass pollen (area under the curve: 0.82; *p* < 0.01; best cut-off ≥7.25%, sensitivity 70.5%, specificity: 90.9%). These predictive methods are important because they let us optimize time and resources in the work-up and management of our patients.

De Carli et al. provided a comprehensive and in-depth review of the molecular mechanisms underlying AR, with a particular focus on sex-related differences. For example, females affected by AR appear to exhibit higher levels of IL-4 and IL-13 than males, and these cytokines are indicative of a classical type-2 phenotype. Yet females show a lower abundance of eosinophils in nasal fluids, and these cells are the hallmark of T2-inflammation. Despite this emerging evidence, the relationship between AR and CRS remains complex and somewhat ambiguous, with AR now recognized as a potential risk factor only for certain subtypes of CRS. Finally, the potential role of AIT in the prevention of future development of CRS and asthma is another area of active investigation, with the impact of sexual dimorphism still to be fully explored.

Kumar et al. at the Mayo Clinic have conducted a systematic review examining the role of viruses in the pathogenesis of CRS. While rhinoviruses (among other species) are well-known causes of acute rhinosinusitis, emerging preclinical and clinical data suggest these pathogens may also contribute to the initiation, exacerbation, and persistence of CRS-related immune dysregulation. Some viruses may drive the inflammatory response —particularly in genetically susceptible individuals— toward type 2 phenotypes, and ultimately lead to the development of nasal polyps. If further validated, this theory could have significant therapeutic implications for CRS treatment and prevention.

As a last contribution, Wang et al. used robust genetic methods, including Mendelian randomization analysis and linkage disequilibrium score, in order to investigate possible causal associations between autoimmune disorders and CRS. By exploiting large genome-wide association study data from several databases, they found a significant relationship between CRS and various autoimmune diseases, including AR and asthma, but also with rheumatoid arthritis or immune-mediated hypothyroidism or type 1 diabetes mellitus. Furthermore, the authors found some loci in three chromosomes to be commonly associated with these conditions. Their findings support the notion that a shared genetic predisposition may underlie the persistence of these disorders, independently of the initial trigger.

In conclusion, the topic of the immunological mechanisms of many ear, nose, and throat disorders is far to be fully explored but the future looks promising. The availability of personalized molecular testing is soon to revolutionize our approach to CRS or tonsillitis. Moreover, while current treatments often rely on symptomatic and long-term therapies (e.g., intranasal topical steroids), the advent of monoclonal antibodies is beginning to shift this paradigm, especially for CRS with nasal polyps. As we continue to explore the fundamental immunopathological mechanisms of otolaryngological disorders, achieving rapid clinical remission and potentially discovering effective cures for these conditions may soon become a reality.
